# Porphyran From *Porphyra haitanensis* Alleviates Obesity by Reducing Lipid Accumulation and Modulating gut Microbiota Homeostasis

**DOI:** 10.3389/fphar.2022.942143

**Published:** 2022-07-25

**Authors:** Xueliang Wang, Juqin Dong, Wei Liang, Yi Fang, Meinong Liang, Lixia Xu, Wuyang Sun, Xiaoxing Li

**Affiliations:** ^1^ Department of Oncology, The First Affiliated Hospital, Sun Yat-sen University, Guangzhou, China; ^2^ Institute of Precision Medicine, The First Affiliated Hospital, Sun Yat-Sen University, Guangzhou, China; ^3^ School of Petrochemical Engineering and Environment, Zhejiang Ocean University, Zhoushan, China

**Keywords:** signaling pathway, lipid accumulation, colonic microbiota, obesity, porphyran from *Porphyra haitanensis*

## Abstract

Porphyran possesses various activities, while the effects of the porphyran from *Porphyra haitanensis* (PPH) on obesity are rarely reported. In this study, C57BL/6J male mice were fed with HFD combined with PPH gavage (50 mg/kg/d) for 16 weeks, and body weight was measured once a week. After that, serum, adipose, and liver tissues were collected for physiological and biochemical analyses. Our research indicated that PPH treatment alleviated obesity in HFD-fed mice. PPH alleviated fat accumulation in serum, liver, and adipose tissues. In addition, PPH activated the AMPK-HSL/ACC pathway in epididymal adipose tissue to reduce lipid accumulation. Moreover, PPH turned white adipose into brown and activated the PGC 1α-UCP 1-mitochondrial pathway in scapular adipose tissue to generate more heat. Interestingly, PPH regulated colonic microbiota homeostasis in obese mice, including significant elevation of *Roseburia* and *Eubacterium* and marked reduction of *Helicobacter*. Moreover, Spearman’s correlation analysis demonstrated that regulation of gut microbiota can decrease lipid accumulation. In summary, our study illustrated that PPH possesses the potential to be developed as an anti-obesity agent.

## 1 Introduction

Obesity is a growing epidemic worldwide and developed into the most significant contributor to health threats (Ogden et al., 2007). In particular, obesity is closely associated with diabetes, metabolic syndrome, non-alcoholic fatty liver disease (NAFLD), and even cancer (Kopelman, 2000), owing to the toxic effects of ectopic accumulation of lipids (Butler et al., 2006). At present, approaches for obesity treatment include drug modulation, surgery, and diet regulation (Plourde, 2000). However, safe and effective weight loss therapies are still of great need so far.

Recently, dietary polysaccharides from seaweed have been demonstrated to confer positive effects in managing metabolic diseases such as obesity (Ahmadi et al., 2017). Porphyran from *Porphyra* is a sulfated polysaccharide that comprises cold-water-soluble portion of the cell wall (Zhang et al., 2004). As a functional seaweed polysaccharide, porphyran from various sources exhibits a series of biological activities, including antioxidant (porphyran from *Porphyra haitanensis*) (Wang et al., 2017), anti-inflammatory (porphyran from *Porphyra vietnamensis*) (Bhatia et al., 2015), immunomodulatory (porphyran from *Porphyra yezoensis*) (Wang et al., 2020), anticancer (porphyran from *Porphyra haitanensis*) (Kwon and Nam, 2007), anti-aging (porphyran from *Porphyra haitanensis*) (Zhang et al., 2018), hypoglycemic (porphyran from *Porphyra haitanensis*) (Cao et al., 2016), and hypolipidemic effects (Hoang et al., 2015).

Numerous studies demonstrated that intestinal microbiota can effectively regulate nutrition and energy metabolism (Sommer and Bäckhed, 2013), which has monumental importance for the regulation of obesity and other metabolic diseases (Kallus and Brandt, 2012). A high-fat diet (HFD) induced microbiome dysbiosis with a decrease in bacterial diversity (Kong et al., 2019). Gut microflora dysbiosis and distinct metabolites may induce obesity (Turnbaugh and Gordon, 2009). Indeed, certain compositions of the gut microbiota may predispose to store excess calories in adipose tissue (Bäckhed et al., 2005). Also, we previously reported that porphyran from *Porphyra yezoensis*–derived oligosaccharides alleviates NAFLD and reshaped the cecal microbiota (Wang et al., 2021). However, effects of porphyran from *Porphyra haitanensis* (PPH) on alleviating obesity and its effect on intestinal microecology were not clear until now. In present study, we first investigated the anti-obesity function of PPH and its underlying mechanism.

## 2 Results

### 2.1 Effects of Porphyran From *Porphyra haitanensis* on Alleviating High-Fat Diet–Induced Obesity

**FIGURE 1:**
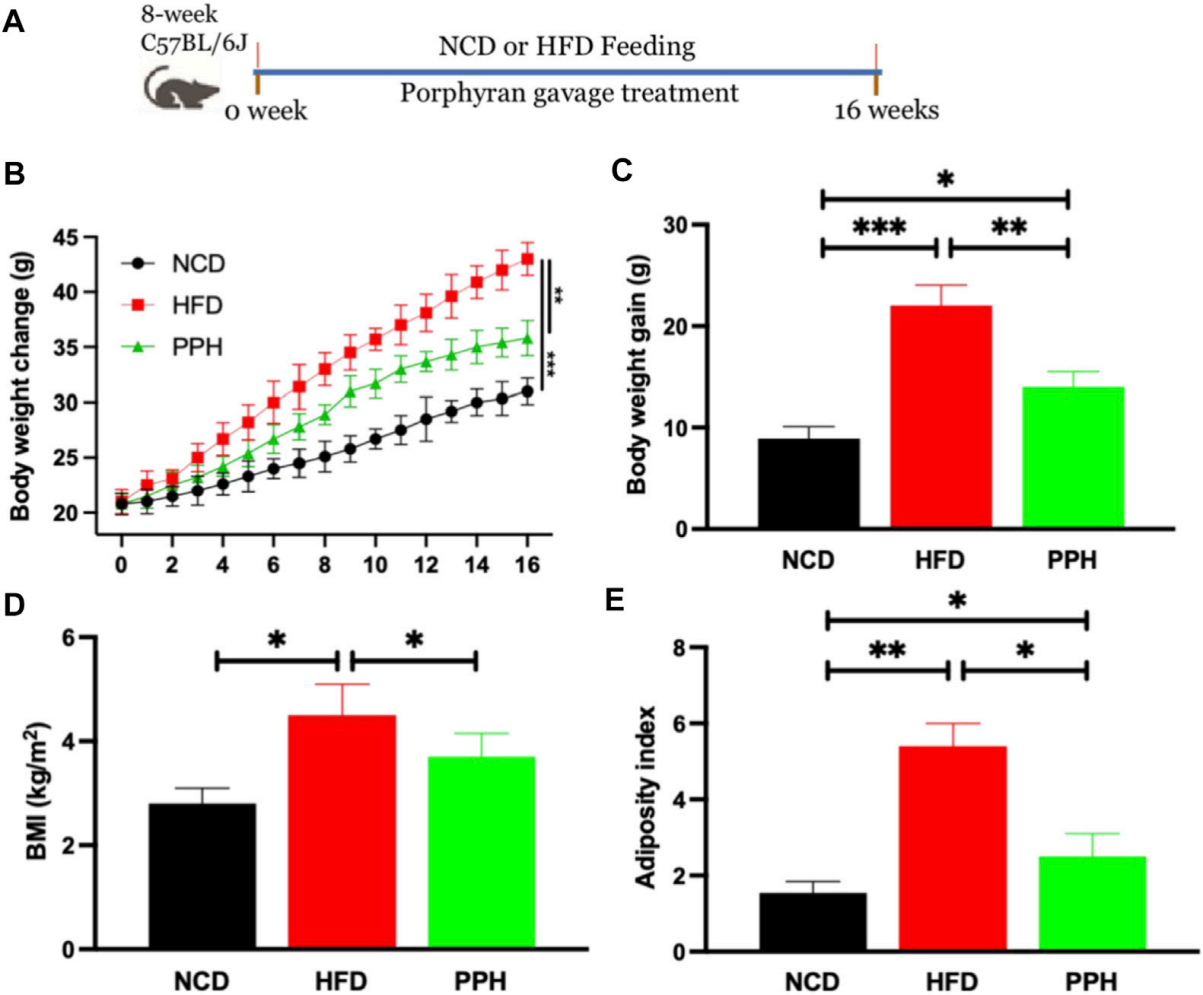
PPH alleviated HFD-induced obesity in mice. Schematic diagram of the animal experiment **(A)**; body weight change **(B)**; body weight gain **(C)**; BMI **(D)**, calculated by body weight/height^2^; adiposity index **(E)**. *p < 0.05, **p < 0.01, and ***p < 0.001.

The animal experiment was designed as shown in Figure 1A. After the 16-weeks feeding trial, body weight of the HFD group was markedly elevated when compared with the NCD-fed group (*p* < 0.001), while PPH administration markedly reversed this adverse situation (*p* < 0.01) (Figure 1B). HFD induced much more weight gain than the NCD group (*p* < 0.001) (Figure 1C), while PPH treatment conspicuously prevented body weight gain (*p* < 0.01) (Figure 1C). Moreover, PPH notably prevented body mass index (BMI) and adiposity index increasement compared with the HFD group (Figures 1D,E) (*p* < 0.05). The abovementioned results prove that PPH can effectively relieve obesity and decrease adiposity index, which may be beneficial for relieving HFD-induced obesity.

### 2.2 Effects of Porphyran From *Porphyra haitanensis* on Reducing Lipid Accumulation in Liver

**FIGURE 2:**
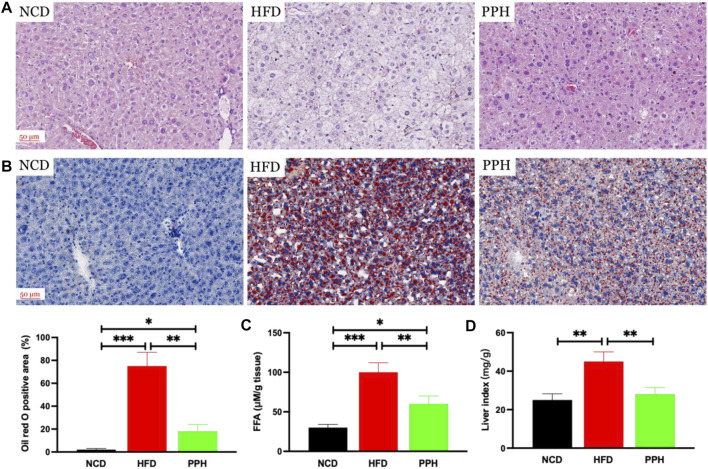
PPH decreased lipid accumulation in liver. Hematoxylin and eosin **(H,E)** staining **(A)**; Oil red O staining **(B)**; FFA **(C)**; liver index **(D)**, calculated by liver weight (mg)/body weight. *p < 0.05, **p < 0.01, and ***p < 0.001.

It has been demonstrated that obesity can exacerbate lipid accumulation in the liver (Prodanović et al., 2016). Histological analysis of the liver tissue was conducted to evaluate the effects of PPH on alleviating hepatic steatosis. We observed that the hepatic lobule structure was clear and complete in the NCD group (Figure 2A). However, the liver tissue was infiltrated with white spots, confirming injury of hepatic cells in the HFD group. Moreover, large areas of necrosis in the central parts of the hepatic lobules were observed, which were characterized by dissolution and disappearance of the nuclei in the HFD group (Figure 2A). After PPH treatment, the abovementioned state was markedly reversed compared with the HFD group and tended to be normal (Figure 2A). Moreover, Oil Red O staining demonstrated that excess lipid accumulation was observed in the HFD group, while PPH treatment significantly decreased the lipid accumulation (*p* < 0.01) (Figure 2B), free fat acid (FFA) (*p* < 0.01) (Figure 2C), and the liver index (*p* < 0.01) (Figure 2D). In general, the abovementioned results indicate that PPH can suppress lipid accumulation in liver to produce an anti-obesity effect.

### 2.3 Effects of Porphyran From *Porphyra haitanensis* on Inhibiting Lipid Accumulation in White Adipose Tissue

**FIGURE 3:**
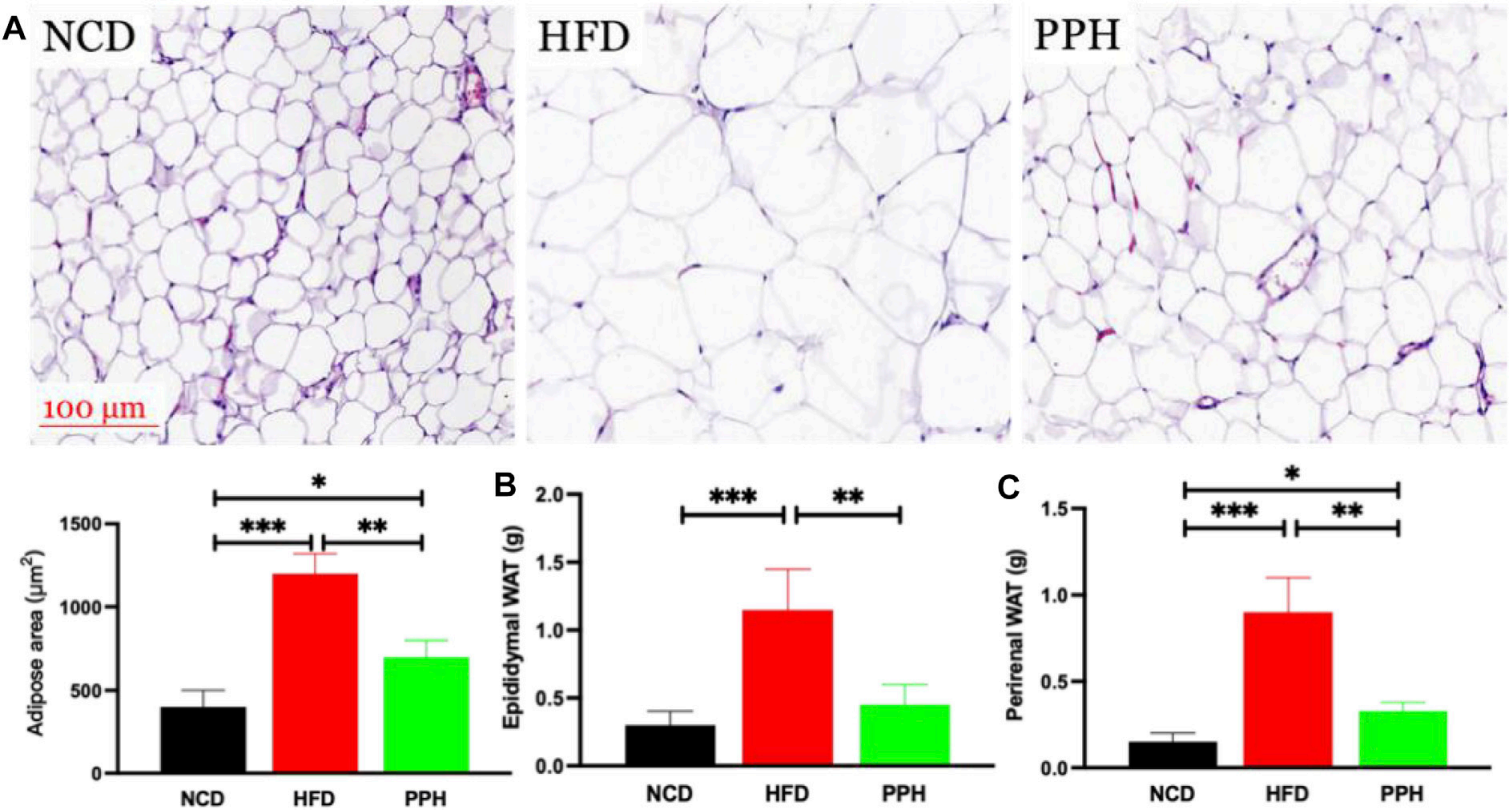
PPH decreased fat accumulation in WAT. H&E staining of epididymal WAT **(A)**; epididymal **(B)** and perirenal WAT weights **(C)**. *p < 0.05, **p < 0.01, and ***p < 0.001.

vWhite adipose tissue (WAT) distribution markedly affects metabolic disease, and increased abdominal and visceral WAT is associated with elevated danger of obesity (Cummins et al., 2014). The major role of WAT is to store superfluous cholesterol and triglycerides (TGs), which is closely related to obesity (Vázquez-Vela et al., 2008). As shown in Figure 3A, the size of the fat bubble was significantly decreased in HFD mice treated with PPH. In addition, PPH intake markedly reduced the epididymal and perirenal WAT weights (Figures 3B,C) (*p* < 0.001) compared with the HFD group. The abovementioned findings indicate that PPH can alleviate obesity by reducing WAT fat accumulation.

### 2.4 Effects of Porphyran From *Porphyra haitanensis* on the 5‘-Monophosphate-Activated Protein Kinase-Hormone-Sensitive Lipase/Acetyl-CoA Carboxylase Signaling Pathway in White Adipose Tissue

**FIGURE 4:**
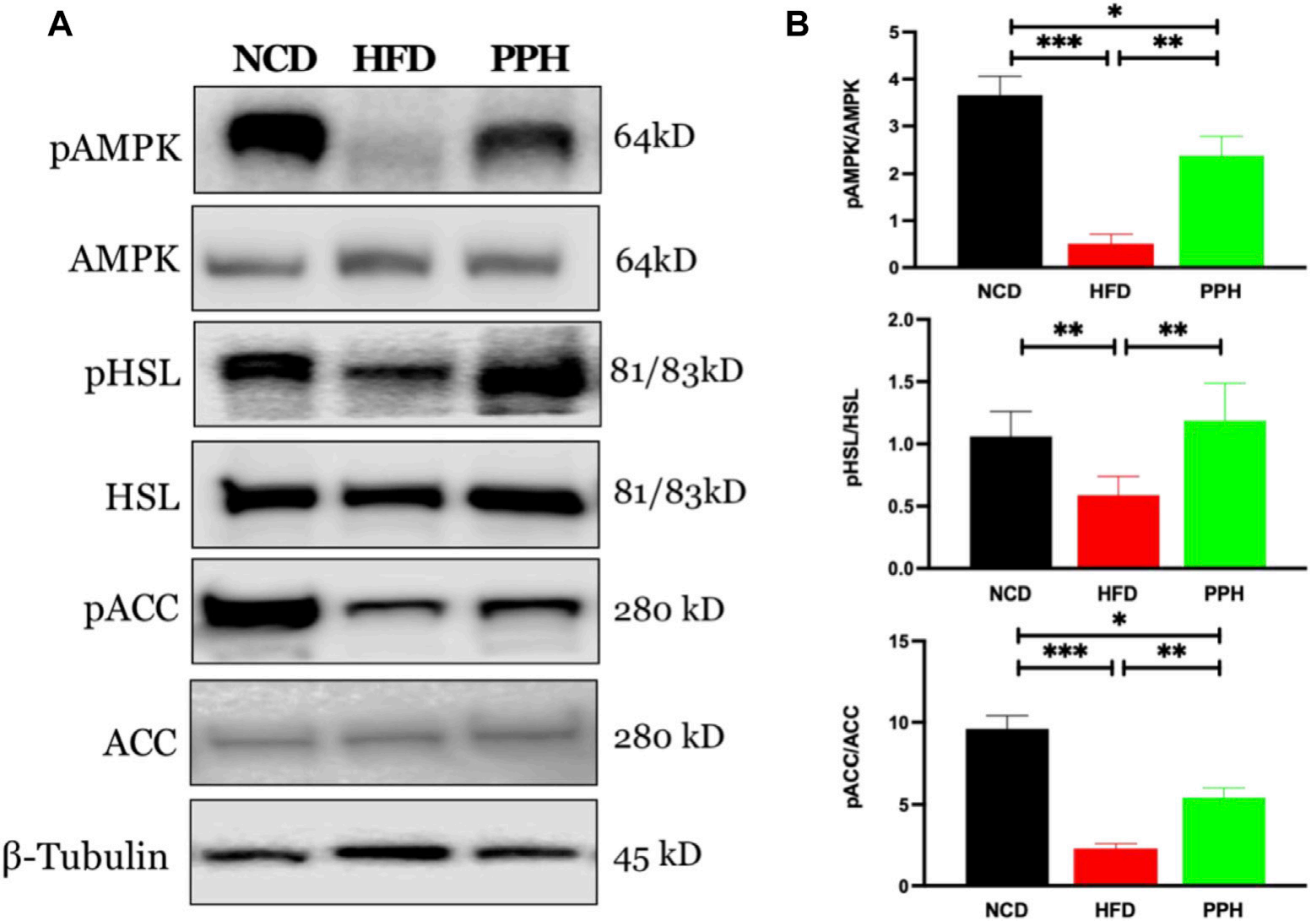
Effects of PPH on activating AMPK signaling pathway in WAT. Western blotting analyses of pAMPK, pHSL, and pACC protein expression in WAT **(A)**; quantification of the pAMPK/AMPK, pHSL/HSL, and pACC/ACC ratios **(B)**. *p < 0.05, **p < 0.01, and ***p < 0.001.

Activated adenosine 5‘-monophosphate-activated protein kinase (AMPK) can limit hepatic triglyceride accumulation (O’Neill et al., 2013). Thus, we further analyzed whether PPH administration could regulate WAT fat metabolism via the AMPK signaling pathway. As shown in Figure 4, the level of pAMPK was markedly reduced in HFD-fed mice compared with the NCD-fed mice (*p* < 0.001). Meanwhile, PPH gavage administration notably increased the activity of AMPK and regulated its downstream proteins, including increasing the levels of phosphorylated hormone-sensitive lipase (pHSL) and phosphorylated acetyl-CoA carboxylase (pACC) (*p* < 0.01). Thus, supplementation of PPH in obese mice can effectively decrease lipid accumulation in WAT by activating the AMPK-HSL/ACC signaling pathway.

### 2.5 Effects of Porphyran From *Porphyra haitanensis* on Reversing Brown to White Conversion in the Scapular Adipose Tissue

**FIGURE 5:**
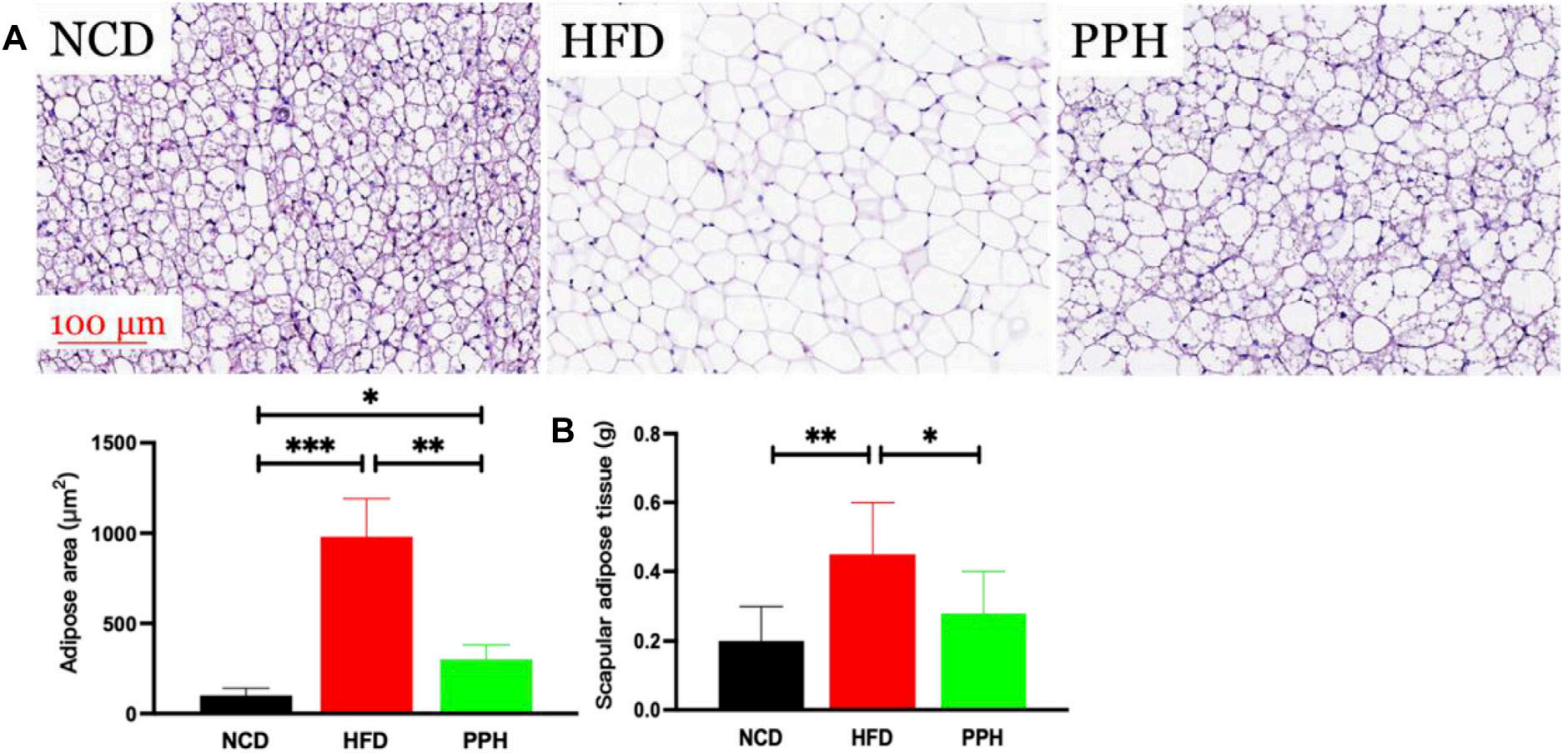
PPH alleviated scapular fat turned into WAT conversion in scapula. H&E staining of scapular adipose tissue **(A)** and scapular adipose tissue weight **(B)**. *p < 0.05, **p < 0.01, and ***p < 0.001.

Scapular adipose mainly comprises brown adipose tissue (BAT) in the lean population, and the thermogenic capacity of scapular fat makes it an attractive therapeutic target for inducing weight loss through energy expenditure (Cypess and Kahn, 2010). The main role of scapular fat is to convert stored energy into heat, which is closely related to obesity (Himms-Hagen, 1989). As shown in Figure 5A, the fat bubble size was notably increased (*p* < 0.001), and the scapular adipose tissue turned into WAT in HFD mice, while PPH treatment notably diminished the fat bubble size (*p* < 0.01) and induced a white-to-brown fat conversion. In addition, PPH intake markedly reduced the scapular fat weight (*p* < 0.05) compared with the HFD-fed mice (Figure 5B). The abovementioned results indicated that PPH can play an anti-obesity role by effectively conducting white-to-brown fat conversion in HFD-fed mice.

### 2.6 Effects of Porphyran From *Porphyra haitanensis* on the Peroxisome Proliferator–Activated Receptor Coactivator 1α-Uncoupling Protein 1-Mitochondria Signaling Pathway in Scapular Adipose Tissue

**FIGURE 6:**
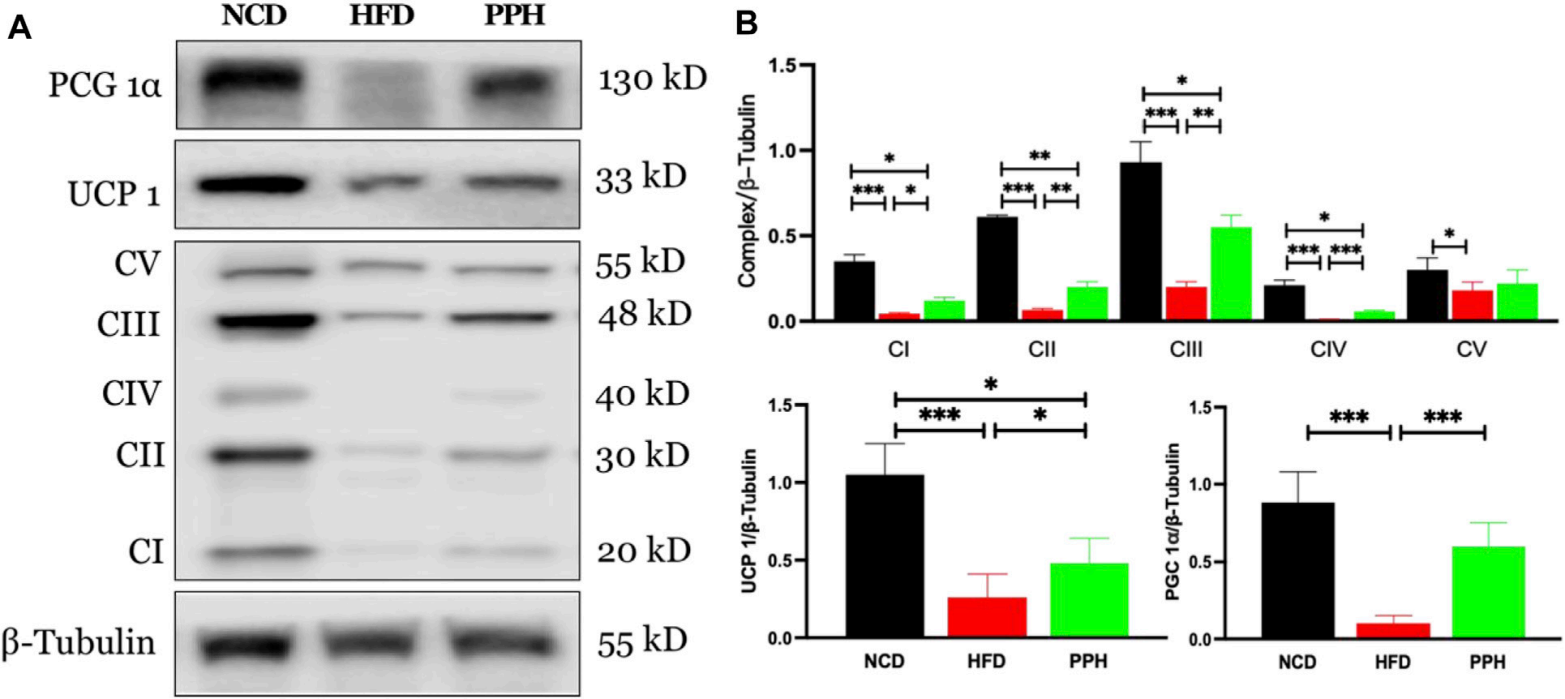
Effects of PPH on activating the mitochondrion-related signaling pathway in scapular adipose tissue. Western blotting analyses of PGC 1α, UCP 1, and mitochondria complex protein expression **(A)**; Quantification of the PGC 1α/β-Tubulin ratio, UCP 1/β-Tubulin ratio, and mitochondria complex/β-Tubulin ratio **(B)**. CI: complex I; CII: complex II; CIII: complex III; CIV: complex IV; CV: complex V. *p < 0.05, **p < 0.01, and ***p < 0.001.

Scapular adipose tissue is rich in mitochondria, which are the energy factories of cells and can easily burn fat to release heat (Zingaretti et al., 2009). Mitochondria in brown adipose tissue produce more heat. Uncoupling protein 1 (UCP1) is rich in mitochondria and is a key to carry out mitochondrial thermogenesis (Muraoka et al., 2009). As shown in Figure 6A, PPH administration increased the expression of peroxisome proliferator–activated receptor coactivator 1α (PGC 1α) (*p* < 0.001) and its downstream target protein UCP 1 (*p* < 0.001) compared with the HFD feeding mice. In addition, PPH treatment increased the expression of mitochondrial respiratory chain complexes I (*p* < 0.05), II (*p* < 0.01), III (*p* < 0.01), and IV (*p* < 0.001) compared with the HFD one, realizing the effect of increasing heat production. And finally, PPH reduces the accumulation of fat in BAT, which has an effect on weight loss.

### 2.7 Effects of Porphyran From *Porphyra haitanensis* on Alleviating Hyperlipidemia in Obese Mice

**FIGURE 7:**
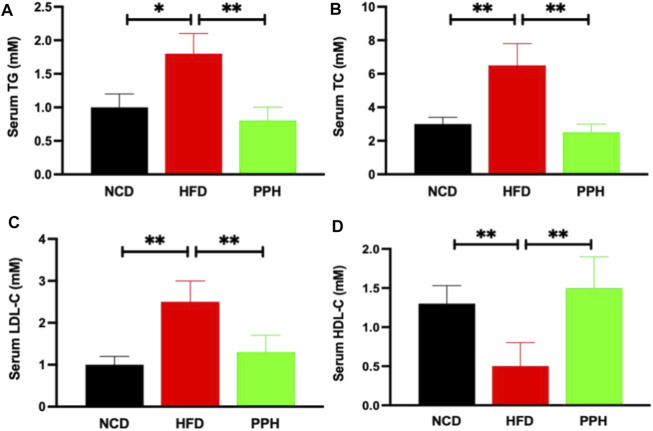
Effects of PPH on serum lipids in obese mice. Serum triglyceride (TG) **(A)**, total cholesterol (TC) **(B)**, low-density lipoprotein cholesterol (LDL-C) **(C)**, and high-density lipoprotein cholesterol (HDL-C) **(D)** levels. *p < 0.05, **p < 0.01.

Obesity can significantly exacerbate the progression of hyperlipidemia (Sullivan et al., 2008), which is a primary health risk factor induced by obesity (Charlton, 2009). HFD can markedly elevated the contents of TG (*p* < 0.05) and TC (*p* < 0.01) in serum compared with the NCD group (Figures 7A,B). After PPH treatment, the TG and TC levels in serum were notably lower than those in the HFD group (*p* < 0.01) (Figures 7A,B). Interestingly, LDL-C decreased, while HDL-C increased in serum after the PPH treatment compared with the HFD one (Figures 7C,D) (*p* < 0.01). In short, the abovementioned data indicate that PPH attenuates serological lipid accumulation in obese mice.

### 2.8 Effects of Porphyran From *Porphyra haitanensis* on Modulating Colonic Microbiota in Obese Mice

**FIGURE 8:**
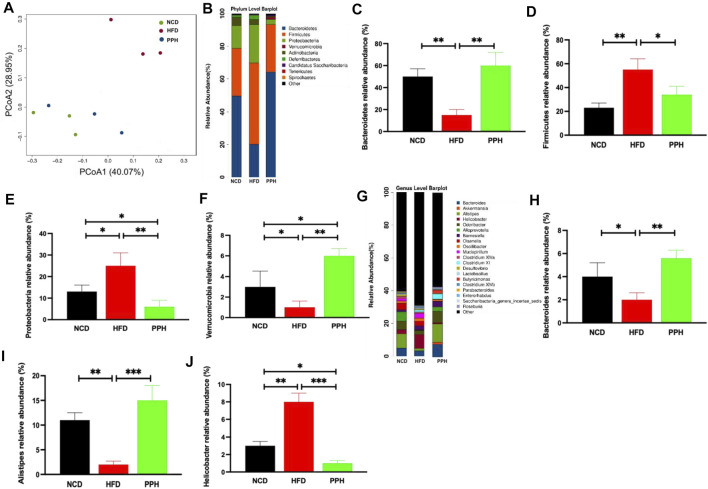
Effects of PPH on regulating colonic microbiota in obese mice. PCoA plot of colonic microbiota based on a weighted UniFrac metric **(A)**; effects of PPH on the relative abundance of gut microbiota at the phylum level **(B)**, such as Bacteroidetes **(C)**, Firmicutes **(D)**, Proteobacteria **(E)**, and Verrucomicrobia **(F)**; Effects of PPH on the relative abundance of gut microbiota at the genus level **(G)**, such as *Bacteroides* **(H)**, *Alistipes* **(I)**, and *Helicobacter* **(J)**. *p < 0.05, **p < 0.01, and ***p < 0.001.

Obesity was characterized by gut microbiota disorder (DiBaise et al., 2008). Also, we demonstrated the effects of PPH on colonic microbiota homeostasis in HFD-fed mice. PCoA analysis indicated that HFD-fed one can markedly change the microbiota composition in the colon compared with the NCD-fed one, which was completely separated (Figure 8A). However, PPH treatment markedly altered the colonic microbiota distribution in mice with HFD feeding, which demonstrated that PPH can be remodeling the microbiota community in the colon (Figure 8A). In addition, the PPH treatment significantly altered the microbiota component at the phylum (e.g., increasing the relative abundances of Bacteroidetes, Proteobacteria, and Verrucomicrobia, while decreasing Firmicutes, Figures 8C–F) and genus level (e.g., increasing the relative abundances of *Bacteroides* and *Alistipes*, while decreasing *Helicobacter*, Figures 8H–J) (Figures 8B,C), which were more similar to that of the NCD group. Altogether, these data demonstrate that colonic micro-ecosystem in obese mice can be effectively regulated by PPH treatment.

### 2.9 Porphyran From *Porphyra haitanensis* Treatment Induced Colonic Microbiota Phylotype Changes

**FIGURE 9:**
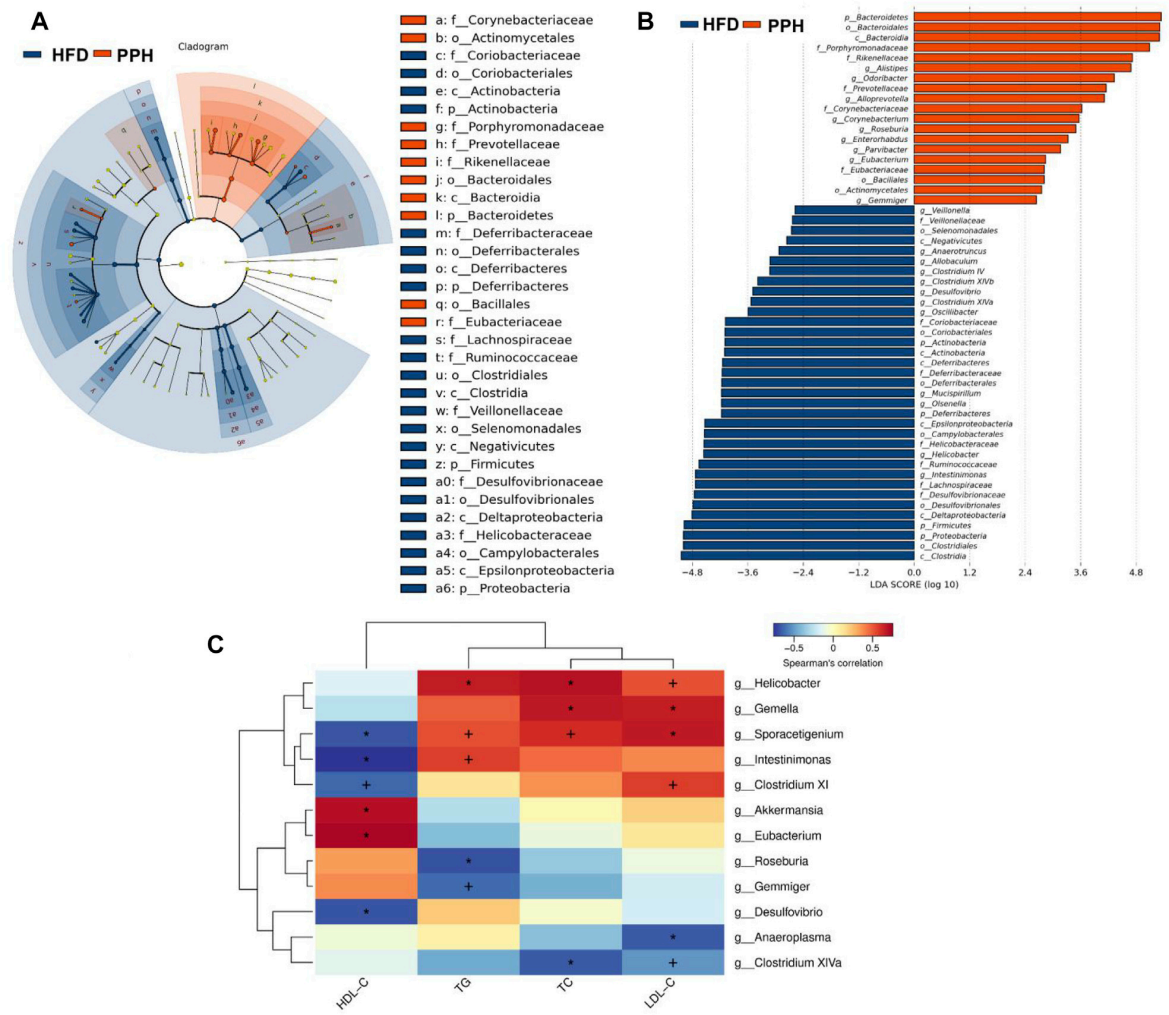
Effects of PPH treatment on phylotypes of colonic microbiota and its correlation with physiological parameters. LEfSe comparison of colonic microbiota between the HFD and the PPH group: Taxa enriched in the HFD group (dark blue) and PPH group (orange) **(A)**; only those meeting the LDA score threshold >2 are listed **(B)**. Heat map of Spearman’s correlations between colonic bacteria and TC, LDL-C, TG, and HDL-C **(C)**. Correlation between biomarkers and microbial species was visualized by color depth: the darker the color, the higher the relevance. + *p* < 0.05 and *p < 0.01.

To clarify the effects of PPH supplement on the regulation of bacterial taxa in the colon, LEfSe analysis was conducted between the HFD and PPH groups. It indicated that PPH decreased the level of Firmicutes, including *Clostridium* XIV, *Veillonella*, and *Helicobacter*, while notably increasing Bacteroidetes, including *Alloprevotella*, *Roseburia*, *Corynebacterium*, and *Alistipes* (Figures 9A,B). In addition, PPH treatment increased the relative abundance of Corynebacteriaceae, Porphyromonadaceae, and Rikenellacea, while a reduction in Veillonellaceae and Desulfovibrionaceae was observed. Moreover, obese mice had a conspicuously elevated abundance of Proteobacteria and Deferribacteres at the phylum level compared with the PPH-treated mice (Figure 9 A and B). In addition, Spearman’s correlation analysis was applied to clarify the relationship between physiological index and bacterial abundance (Figure 9C). At the genus level, *Roseburia* and *Eubacterium* were positively related to HDL-C levels while negatively related to TC, TG, and LDL-C levels. Meanwhile, the effects of Helicobacter were opposite. More importantly, PPH triggered an increase in the relative abundances of *Roseburia* and *Eubacterium* in obese mice, while decreasing the abundances of *Helicobacter*. The abovementioned results showed that alteration in the gut microbiota composition after PPH gavage brings about a lipid level decrease in obese mice.

## 3 Discussion

Effects of the water-soluble polysaccharide from *Porphyra haitanensis* on alleviating obesity were rarely reported. This current study demonstrated that PPH markedly ameliorated HFD-induced obesity, mainly by alleviating lipid accumulation in WAT, scapular adipose tissue, liver, and serum. Moreover, PPH can effectively regulate colonic microbiota dysbiosis, which is related to alleviation of obesity.

AMPK can maintain energy homeostasis, which is regarded as a considerable energy sensor (Horman et al., 2002). Activated AMPK reduces lipid synthesis and accelerates fatty acid oxidation by manipulating its downstream targets such as HMG-CoA reductase and ACC (Lee et al., 2006; Srivastava et al., 2012). In this study, PPH-treatment activated the AMPK-HSL/ACC signaling pathway in WAT compared with the HFD group, thus reducing fat accumulation in WAT.

It has been confirmed that BAT activity is inversely related to BMI and body fat percentage (van Marken Lichtenbelt et al., 2009). BAT is becoming a new target for anti-obesity therapies focusing on increasing thermogenesis (Cypess and Kahn, 2010). PGC 1α and its downstream target UCP-1 in BAT can be activated to modulate mitochondrial and metabolic homeostasis (Golozoubova et al., 2001; Puigserver and Spiegelman, 2003). The impairment of signaling involved in the regulation of PGC 1α causes obesity (Crunkhorn et al., 2007). Also, dietary ingredients have been demonstrated to stimulate BAT function. We confirmed that PPH activated the PGC 1α-UCP 1 signaling pathway and increased the mitochondrial complex activities, which resulted in a dramatic increase in heat production and induced a white-to-brown fat conversion in the scapular fat of obese mice.

Furthermore, gut microbiota served as a vital regulator of lipid metabolism (Sonnenburg and Bäckhed, 2016). Intestinal microecology of the obese population showed decreased Bacteroidetes/Firmicutes ratio, indicating that various gut microbiota compositions can affect energy metabolism (DiBaise et al., 2008). Moreover, fecal microbiota transplantation from obese donors increased body weight gain and BMI (Turnbaugh et al., 2009), which indicated a causal association between microbiota and obesity outcomes (Liou et al., 2013). Interestingly, PPH regulated colonic microbiota homeostasis in obese mice. PPH reduced the level of Firmicutes, including *Helicobacte*, *Veillonella*, and *Clostridium* XIV, while remarkedly elevating Bacteroidetes, such as *Alloprevotella*, *Roseburia*, *Corynebacterium*, and *Alistipes*. In addition, Spearman’s correlation analysis indicated that colonic microbiota alteration could decrease lipid accumulation. In general, our present study strengthens the linkage between obesity and gut dysbiosis and illustrated that porphyran from *Porphyra haitanensis* possess the potential to be developed as an anti-obesity agent.

## 4 Materials and Methods

### 4.1 Porphyran From *Porphyra haitanensis* Preparation

PPH was isolated from cold-water-soluble extracts of *Porphyra haitanensis* as previously described (Qiu et al., 2021). In brief, the algae were powdered and treated with 50% ethanol solution to remove excess lipid and then water-soluble polysaccharides were extracted with water [1.5% (w/v)] at room temperature for 10 h. After centrifugation, the supernatant was mixed with double volumes of ethanol. After centrifugation, the precipitate was vacuum-dried to get the PPH.

### 4.2 Animals Experimental Design

Animal experiments were conducted as shown in Figure 1A. Briefly, 8-week-old SPF C57BL/6J mice were purchased from the Gempharmatech Co., Ltd (Nanjing, China). They were raised in individually ventilated cages, and diet was provided *ad libitum*. Mice were divided into three groups (10 per group) after 1 week of acclimation. Mice in the NCD group were fed a normal chow diet (D12450B, Research Diets Inc., New Brunswick, NJ, United States), while the HFD and the PPH groups were fed a high-fat diet (HFD) (D12492, Research Diets Inc., New Brunswick, NJ, United States) for 16 weeks to induce obesity. Meanwhile, PPH group received 50 mg/kg/d PPH by gavage, while NCD and HFD groups were given aliquots of phosphate-buffered saline (PBS). At end of the trial, mice were euthanized after starving overnight. Blood was collected and then centrifuged at 2,000 g for 15 m to obtain serum.

### 4.3 Histological Assessment

Liver tissues, epididymal fat, perirenal fat, and scapular fat tissues were collected and weighed. Tissues were stored for Oil Red O staining and Western blot analysis at −80°C. Partial tissues were fixed in 4% paraformaldehyde solution immediately. After dehydration and embedding, tissues were cut into 4-μm-thick sections and analyzed by H&E or Oil Red O (Wang et al., 2021; Wang et al., 2022). Quantity was analyzed using ImageJ software.

### 4.4 Biochemical Assays

TC, TG, FFA, LDL-C, and HDL-C levels were measured by corresponding kits according to the manufacturer’s instructions (F001-1-1, F002-1-1, A042-2-1, A112-1-1, and A113-1-1; Jiancheng Bioengineering Institute, Nanjing, China).

### 4.5 Western Blot

Proteins of adipose tissues were extracted with the Minute Total Protein Extraction Kit (SD-001, Invent Biotechnologies, Eden Prairie, MN, United States). After denaturation with loading buffer at 100°C for 10 m, 40 μg proteins were loaded on 10% SDS-PAGE and then transferred to PVDF membranes and then incubated with primary antibodies directed against AMPKα (2532, CST, Danvers, MA, United States), pAMPK (2531, CST), ACC (3662, CST), pACC (11818, CST), HSL (18381, CST), pHSL (45804, CST), UCP 1 (72298, CST), PGC 1α (2178, CST), and β-Tubulin (2146, CST) at 4°C overnight, while Total OXPHOS Rodent WB antibody Cocktail (ab110413, Abcam, Cambridge, United Kingdom) was used to analyze the relative levels of OXPHOS complexes. After washing with TBST, membranes were incubated with secondary antibodies at room temperature for another 2 h. The Omni-ECL™Femto Light Chemiluminescence Kit (SQ201, Epizyme Biomedical Technology Co., Ltd., Shanghai, China) was used to visualize protein bands. In addition, membranes were stripped with Minute WB stripping solution (WA-003, Invent Biotechnologies, Eden Prairie, MN, United States). ImageJ software was used for densitometry analysis.

### 4.6 16S rDNA Sequencing

The QIAamp DNA Stool Mini Kit (51604, Qiagen, Hilden, Germany) was used to extract bacterial DNA from colonic contents. Then, universal primers (341F and 806R) were utilized to amplify the bacterial 16S rDNA gene. Then, amplicons were sequenced on an Illumina Miseq PE250 by Biozeron (Biozeron, Shanghai, China), and OTUs were generated by clustering at 3% difference. In addition, microbial taxa differences between various groups were characterized by linear discriminant analysis (LDA) effect size (LEfSe). Heat maps were analyzed using R Cytoscape, which showed the relationship between physiological index and colonic gut microbiota.

### 4.7 Statistical Analysis

Differences between groups were analyzed by GraphPad Prism (GraphPad Software Inc., San Diego, CA, United States). Data are presented as the mean ± standard error of the mean (SEM). Also, one-way ANOVA was applied to analyze multiple comparisons. The difference was considered to be statistically significant while *p* < 0.05.

## Data Availability

The datasets presented in this study can be found in online repositories. The names of the repository/repositories and accession number(s) can be found below: https://www.ncbi.nlm.nih.gov/sra/PRJNA836424.

## Abbreviations

PPH, porphyran from *Porphyra haitanensis*; PBS, phosphate-buffered saline; HFD, high-fat diet; NAFLD, non-alcoholic fatty liver disease; NCD, normal chow diet; WAT, white adipose tissue; BAT, brown adipose tissue; BMI, body mass index; TG, triglyceride; FFA, free fatty acid; TC, total cholesterol; HDL-C, high-density lipoprotein cholesterol; LDL-C, low-density lipoprotein cholesterol; H&E, hematoxylin, and eosin; AMPK, 5‘-monophosphate-activated protein kinase; HSL, hormone-sensitive lipase; ACC, acetyl-CoA carboxylase; PGC 1α, peroxisome proliferator–activated receptor coactivator 1α; UCP 1, uncoupling protein 1; LDA, linear discriminant analysis; LEfSe, linear discriminant analysis effect size.
